# Diagnostic challenges of prolonged post-treatment clearance of *Plasmodium* nucleic acids in a pre-transplant autosplenectomized patient with sickle cell disease

**DOI:** 10.1186/s12936-017-2152-x

**Published:** 2018-01-10

**Authors:** Paul M. Luethy, Sean C. Murphy, Annette M. Seilie, Yingda L. Xie, Chuen-Yen Lau, John F. Tisdale, Matthew M. Hsieh, Jessica L. Reinhardt, Anna F. Lau, Gary A. Fahle

**Affiliations:** 1 Microbiology Service, Department of Laboratory Medicine, National Institutes of Health, 10 Center Drive, Bldg. 10, 2C306, Bethesda, MD 20892-1508 USA; 2 Department of Laboratory Medicine, University of Washington, Seattle, WA 98109 USA; 3 Department of Microbiology, University of Washington, Seattle, WA 98109 USA; 4 The Center for Emerging and Re-emerging Infectious Diseases, University of Washington, Seattle, WA 98109 USA; 5 National Institute of Allergy and Infectious Diseases, National Institutes of Health, Bethesda, MD 20892 USA; 6 Molecular and Clinical Hematology Branch, National Heart, Lung, and Blood Institute, National Institutes of Health, Bethesda, MD 20892 USA

**Keywords:** *Plasmodium falciparum*, Sickle cell disease, Autosplenectomy, PCR, RT-PCR, 18S rRNA, NAAT, Nucleic acid amplification test

## Abstract

**Background:**

Autosplenectomy, as a result of sickle cell disease, is an important risk factor for severe malaria. While molecular methods are helpful in providing rapid and accurate infection detection and species identification, the effect of hyposplenism on result interpretation during the course of infection should be carefully considered.

**Case presentation:**

A 32-year old autosplenectomized Nigerian male with severe sickle cell disease was referred to the National Institutes of Health for allogenic hematopoietic stem cell transplant. Despite testing negative for malaria by both smear and PCR 2 weeks after arrival in the USA, the patient developed fever and diffuse bilateral lower rib cage and upper abdominal pain 2 weeks later and subsequently tested positive for *Plasmodium falciparum*. Parasitaemia was tracked over time by microscopy and nucleic acid tests to evaluate the therapeutic response in the setting of hyposplenism. The patient showed prompt resolution of patent infection by microscopy but remained positive by molecular methods for > 30 days after treatment initiation.

**Conclusion:**

While molecular testing can provide sensitive *Plasmodium* nucleic acid detection, the persistence of *Plasmodium* nucleic acids following adequate treatment in functionally asplenic patients can lead to a diagnostic dilemma. In such patients, clinical response and peripheral blood smears should guide patient management following treatment. Nonetheless, in pre-transplant patients at high-risk for pre-existing *Plasmodium* infections, highly sensitive molecular assays can be useful to rule out infection prior to transplantation.

## Background

Malaria remains a prominent cause of infection and mortality worldwide with ~ 212 million new infections in 2015 and almost 429,000 reported deaths [1]. Malaria transmission has been identified in 91 countries, with 90% of all cases and 92% of deaths occurring in sub-Saharan Africa. Sickle cell disease (SCD), which leads to haemolytic anaemia, acute vaso-occlusive events, and chronic end-organ damage, is also a serious cause of childhood mortality in sub-Saharan Africa [2]. Studies have shown that a heterozygous mutation in the β-globin allele can confer protection from severe malaria compared with the homozygous mutation due to reduced cytoadherence, increased sickling with subsequent changes in host cell biochemistry, and increased splenic clearance [3–6]. The presence of heterozygous sickle cell trait, however, does not prevent infection.

This case concerns a patient with SCD and autosplenectomy, who travelled from Nigeria to the USA to undergo allogenic haematopoietic stem cell transplantation (HSCT). Discussion points include the conundrum of initial negative malaria screening results, a subsequent diagnosis of *Plasmodium falciparum* malaria, and clinical considerations when faced with conflicting diagnostic test results post-treatment due to the persistence of *Plasmodium* nucleic acids in blood samples.

## Case presentation

**Fig. 1:**
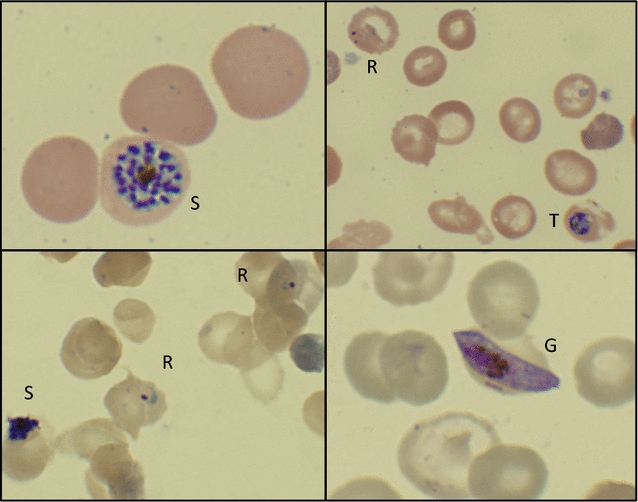
*Plasmodium falciparum* forms observed in stained blood smears. Thin smears of peripheral blood with either Wright Giemsa (top) or Giemsa (bottom) staining. Parasite forms included immature trophozoites (rings, R), mature trophozoites (T), schizonts (S) and gametocytes (G). Pictured fields are representative of samples collected on day 0 (top panels and bottom left) and day 2 (bottom right)

**Fig. 2:**
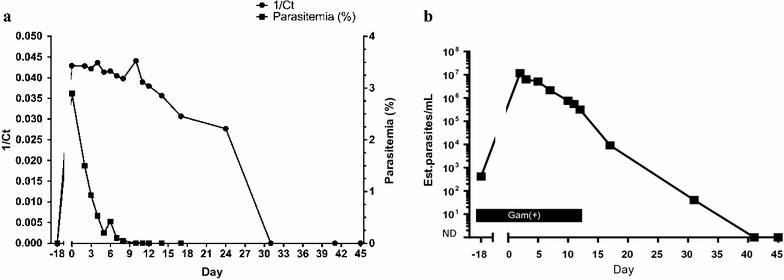
Patient’s course of microscopy and molecular PCR and qRT-PCR assay positivity. **a** Graphical correlation of microscopy-based parasitaemia (%, right axis) with detection of Plasmodium DNA by a multiplex malaria DNA PCR assay (represented as 1/cycle threshold (1/Ct), left axis) from days-18 to 45. **b** Retrospective qRT-PCR performed on samples from days-18 to 45. Estimated (Est.) parasites/mL is based on the pan-Plasmodium 18S rRNA channel; the black bar shows the date range where gametocyte mRNA was also qualitatively detected. Parasite burden on days 41 and 45 was below the limit of detection (not detected [ND] < 20 parasites/mL)

This is a case of a 32-year old Nigerian man with homozygous SCD. Despite receipt of hydroxyurea, he continued to experience monthly priapism and three to four hospitalizations for vaso-occlusive pain crises per year. He reported a history of several malaria infections including a severe episode that led to acute chest syndrome and heart failure requiring intensive care unit monitoring and red cell transfusions. The patient travelled from Nigeria to the USA (arriving on day-32) in preparation to receive a non-myeloablative allogeneic HSCT from his fully human leukocyte antigen-matched brother. As part of his pre-transplant evaluation on day-18 (2 weeks after entering the USA from Nigeria), blood was obtained for malaria thick and thin smears and for *Plasmodium* multiplex PCR; all testing was reported as negative. On day-4, the patient developed fever as well as diffuse bilateral lower rib cage and upper abdominal pain. He was admitted on day-1 for a vaso-occlusive pain crisis. On day 0, the patient developed fevers to 39.1°C and a decrease in absolute haemoglobin from 8.2 to 5.8 g/dL. Possible intraerythrocytic parasites were observed initially from routine cell count and differential smears, and repeat malaria blood smears were positive for immature (ring form) and mature trophozoites and schizonts of *P. falciparum* at 2.9% parasitaemia (Fig. 1). Parallel testing with a multiplex malaria PCR ([7–9]; limit of detection (LOD) defined as 700 parasites/mL of whole blood) was also positive (Fig. 2a). Treatment with artemether–lumefantrine (20/120 mg, Coartem^®^) was started on day 1 after consultation with the clinical team and pharmacy. The patient was supported with RBC transfusions and defervesced by day 3 with 0.93% parasitaemia. Between days 5 and 6, his parasitaemia levels rose from 0.20 to 0.42%, respectively (Fig. 2a). In response, he was immediately started on atovaquone/proguanil (Malarone^®^) for 7 days due to concerns for artemether–lumefantrine resistance. Reflex testing by the Centers for Disease Control and Prevention did not reveal genetic markers on the *kelch 13* gene that was consistent with artemisinin resistance. The patient remained clinically well with < 0.05% parasitaemia and was discharged after three doses of atovaquone/proguanil (day 8). Blood smears were negative by day 10, but the multiplex malaria PCR assay remained positive on day 17. Because of his scheduled transplant and slow parasite clearance, he received a course of doxycycline for 3 days. The multiplex malaria PCR assay became negative between days 25 and 31. The patient proceeded with the allogeneic HSCT on day 41 and engrafted fully with donor red cells without recurrence of malaria during the transplant process. He has returned to Nigeria and has remained clinically well.

Retrospective testing was performed on 12 preserved whole blood samples using a quantitative reverse transcription PCR (qRT-PCR) that targets the highly expressed *Plasmodium* 18S rRNAs (LOD: 20 parasites/mL of whole blood [10, 11]). qRT-PCR demonstrated ~ 420 estimated parasites/mL of whole blood in the initial day-18 screening sample that was previously reported negative by peripheral blood smears and multiplex PCR (Fig. 2b). Parasite burden as measured by qRT-PCR decreased over time following treatment with positive results detected more than 1 month after treatment initiation. Additional molecular testing targeting the gametocyte-specific spliced PF3D7_0630000 mRNA [12] showed gametocyte detection on days-18 through 11 (Fig. 2b). Gametocyte load was highest at the peak of infection (days 0–5).

## Discussion and conclusion

Timely detection of *Plasmodium* parasites in peripheral blood specimens is important for effective management of patients with suspected malaria infection [13]. Accurate species level identification along with detailed travel history are critical for determining appropriate anti-malarial therapy. Here, the characteristic microscopic structures of *P. falciparum* (immature trophozoite ring forms and occasional crescent-shaped gametocytes) along with the rarely present mature trophozoites and schizonts were observed (Fig. 1). The latter has been reported in patients without a functioning spleen, which is normally responsible for removing these mature forms [14].

Although serial blood smears and multiplex PCR demonstrated a response to treatment, blood smears correlated better with clinical improvement compared with PCR results (Fig. 2a). Prolonged PCR positivity resulted in two additional treatment courses with atovaquone/proguanil and doxycycline (despite negative blood smears) to ensure complete eradication of *P. falciparum* prior to transplantation. In retrospect, the day-18 sample was positive using a highly sensitive *Plasmodium* 18S rRNA qRT-PCR assay, indicating that the patient was infected prior to leaving Nigeria (Fig. 2b). Thus, while asymptomatic on initial presentation, the patient was incubating an unrecognized *Plasmodium* infection missed by both routine peripheral blood smears and multiplex PCR because the parasite burden (~ 420 parasites/mL) was below the LODs for these assays (5000–20,000 parasites/mL for thick blood smears by an expert reader [15] and 700 parasites/mL for the multiplex PCR). Molecular tests such as the qRT-PCR assay provide estimated parasite densities that correlate with microscopy-based estimates [16]. Five days after treatment initiation, the qRT-PCR assay indicated that the parasite density decreased by > 80%, which corresponded to clinical improvement. Despite a long period of qualitative molecular positivity, quantitative nucleic acid testing that are reflective of estimated parasite densities can help guide patient care.

The presence of mature parasite forms in peripheral blood smears and the delay in PCR clearance after treatment created a diagnostic challenge for clinicians. In this patient, these phenomena were most likely due to biological changes caused by the patient’s underlying SCD. Normally, RBCs harbouring mature trophozoites and schizonts are often sequestered in organ microvasculature [17] due to the translocation of parasite proteins to the RBC surface that bind to ligands on the organ and tissue epithelial cell surface [18]. In individuals with SCD, this surface protein expression is decreased resulting in reduced cytoadherence of infected RBCs [19]. In this patient, however, autosplenectomy due to SCD was most likely the primary driver of this presentation [20–23].

In functionally asplenic patients like the patient presented here, it is likely that *Plasmodium*-derived nucleic acids persist in peripheral blood cells longer than in persons with normal spleens. This can lead to a diagnostic dilemma if molecular tests alone are used to monitor parasite clearance following treatment. In persons with normal splenic function, it is known that molecular assays can remain positive at low-levels following treatment beyond microscopically-apparent clearance of organisms [10]. In controlled human malaria infection (CHMI) studies, ~ 50% of blood smear-positive persons rapidly become RT-PCR negative post-treatment and the others show substantial reductions in RT-PCR-measured parasite load; all subjects were RT-PCR negative 2–3 weeks later [10]. It is possible and likely that molecular positivity resolved earlier than the end of study time point, but no test-of-cure samples were collected during days 6–15 post-treatment [10]. Additional data regarding clearance of *Plasmodium* 18S rRNA is being accrued in multiple ongoing, unpublished CHMI studies (SC Murphy, pers. comm.). Regardless of splenic status, clinicians must consider whether ongoing molecular positivity in a treated patient is due to slower clearance of non-viable parasite-derived nucleic acids or represents a potential ongoing viable infection. Gradually-declining molecular assay-derived measurements of parasite density are most likely representative of non-viable, slow-clearing parasite biomass. In contrast, resurgent or clear positive persistence could represent impending treatment failure, which has been recognized for drug-resistant parasites manifesting as persistent PCR positivity in the week following initial treatment [21]. Thus, additional studies are needed to better understand the duration of molecular test positivity in appropriately-treated patients with or without a functional spleen. This information will be useful for guiding decisions about second-line treatments. In this case study, blood smears corresponded to the patient’s clinical status. In pre-transplant patients with high-risk for pre-existing *Plasmodium* infections, highly sensitive molecular assays such as the ultrasensitive qRT-PCR assay used herein may be useful for ruling out infection prior to transplantation. The LOD, range of species detected, and sample stability should be considered when selecting such tests.

## Abbreviations

SCD: sickle cell disease
HSCT: hematopoietic stem cell transplantation
LOD: limit of detection
Ct: cycle threshold
PCR: polymerase chain reaction
RT-PCR: reverse transcription PCR
rRNA: ribosomal RNA
